# Glioblastoma Proximity to the Lateral Ventricle Alters Neurogenic Cell Populations of the Subventricular Zone

**DOI:** 10.3389/fonc.2021.650316

**Published:** 2021-06-29

**Authors:** Luisina B. Ripari, Emily S. Norton, Raquel Bodoque-Villar, Stephanie Jeanneret, Montserrat Lara-Velazquez, Anna Carrano, Natanael Zarco, Carla A. Vazquez-Ramos, Alfredo Quiñones-Hinojosa, Carlos de la Rosa-Prieto, Hugo Guerrero-Cázares

**Affiliations:** ^1^ Department of Medical Sciences, Facultad de Medicina de Albacete, Universidad de Castilla-La Mancha, Albacete, Spain; ^2^ Department of Neurosurgery, Mayo Clinic, Jacksonville, FL, United States; ^3^ Neuroscience Graduate Program, Mayo Clinic Graduate School of Biomedical Sciences, Jacksonville, FL, United States; ^4^ Regenerative Sciences Training Program, Center for Regenerative Medicine, Mayo Clinic, Jacksonville, FL, United States; ^5^ Translational Research Unit, Hospital General Universitario de Ciudad Real, Ciudad Real, Spain; ^6^ Faculty of Psychology and Sciences of Education, University of Geneva, Geneva, Switzerland

**Keywords:** glioblastoma, subventricular zone (SVZ), lateral ventricle, neural stem cell (NSC), cancer stem cell (CSC), neurogenic niche

## Abstract

Despite current strategies combining surgery, radiation, and chemotherapy, glioblastoma (GBM) is the most common and aggressive malignant primary brain tumor in adults. Tumor location plays a key role in the prognosis of patients, with GBM tumors located in close proximity to the lateral ventricles (LVs) resulting in worse survival expectancy and higher incidence of distal recurrence. Though the reason for worse prognosis in these patients remains unknown, it may be due to proximity to the subventricular zone (SVZ) neurogenic niche contained within the lateral wall of the LVs. We present a novel rodent model to analyze the bidirectional signaling between GBM tumors and cells contained within the SVZ. Patient-derived GBM cells expressing GFP and luciferase were engrafted at locations proximal, intermediate, and distal to the LVs in immunosuppressed mice. Mice were either sacrificed after 4 weeks for immunohistochemical analysis of the tumor and SVZ or maintained for survival analysis. Analysis of the GFP+ tumor bulk revealed that GBM tumors proximal to the LV show increased levels of proliferation and tumor growth than LV-distal counterparts and is accompanied by decreased median survival. Conversely, numbers of innate proliferative cells, neural stem cells (NSCs), migratory cells and progenitors contained within the SVZ are decreased as a result of GBM proximity to the LV. These results indicate that our rodent model is able to accurately recapitulate several of the clinical aspects of LV-associated GBM, including increased tumor growth and decreased median survival. Additionally, we have found the neurogenic and cell division process of the SVZ in these adult mice is negatively influenced according to the presence and proximity of the tumor mass. This model will be invaluable for further investigation into the bidirectional signaling between GBM and the neurogenic cell populations of the SVZ.

## Introduction

Glioblastoma (GBM) is the most frequent and aggressive type of malignant primary brain tumor in adults (1, 2). Patients suffering from GBM have a median survival of approximately 15 months despite advanced therapeutic strategies of combinatorial surgery, chemotherapy, and radiation (3, 4). Interestingly, tumor progression for GBM patients is greatly affected by tumor location. Lateral ventricles (LVs) infiltrating tumors account for over 50% of all GBM patients (5). These LV-contacting tumors result in higher incidence of distant recurrence, as well as larger tumor volume and worse survival expectancy in patients (6–9). Furthermore, GBM patients who receive radiotherapy that includes the ventricular wall ipsilateral to the tumor show increased survival when compared to patients where the ipsilateral ventricular wall is avoided (10), indicating the involvement of LV-derived factors in GBM progression.

The reason for worse patient outcome in these cases is unknown, though could be due in part to contact with the subventricular zone (SVZ) present in the lateral wall of the LV. The SVZ is the largest stem cell niche in the mammalian adult brain, including humans (11–14). In rodents, neural stem cells (NSCs) of the SVZ form new neurons and glia throughout life, differentiating to neuroblasts or glial progenitors that then migrate to their site of terminal differentiation (15–18). Studies have shown a high amount of similarity in the biology of NSCs and stem-like GBM cells, including shared pathways of self-renewal, differentiation, and cell migration (19, 20). Additionally, several studies have identified NSCs of the SVZ as a potential cell-of-origin for GBM, pointing to the potential involvement of NSCs in GBM progression (21–25).

Shared mechanisms between NSCs and GBM support the idea that stem cell-supportive factors contained within the SVZ support the proliferation and stemness of LV-proximal tumors. This may include a bidirectional crosstalk between NSCs/progenitors and GBM cells that leads to changes in SVZ biology and patient outcome. Previous work has shown that cell types within the SVZ, including NSCs and their progeny, are altered in response to GBM in rodents (26). Here, we develop a novel rodent model of LV-proximal GBM and examine the reciprocal relationship between the SVZ and GBM tumors. We particularly focused on cell population and proliferation changes in the SVZ as a consequence of GBM proximity to the LV.

## Materials and Methods

### Experimental Animals

All *in vivo* experiments were approved by the Institutional Animal Care and Use Committee of Mayo Clinic. Mice were housed in a fully AALAC-accredited facility in accordance with all federal and local regulations. Male athymic immunosuppressed J:NU mice (The Jackson Laboratory, strain 007850) were maintained at Mayo Clinic Jacksonville with a 12-hour light-dark cycle and *ad libitum* feeding. Animals were utilized for experiments at an age between 6-8 weeks.

### Primary GBM Cell Xenograft and Euthanasia

We utilized a primary cell line of human GBM cells (GBM1A, also known as line 020913) (27). GBM1A cells were transduced with a GFP-luciferase lentivirus (RediFect™ Red-FLuc-GFP, Perkin Elmer CLS960003) and sorted for GFP positivity. Following cell transduction, intracranial implantation of tumors was performed as previously described (28–31). Briefly, mice were anesthetized and placed in a stereotactic frame. 5.0 x10^5^ GBM1A-GFP luciferase+ cells were injected in 2 μL of DMEM/F12 into the right brain hemisphere. 3 injection sites were established in the following coordinates (in mm relative to bregma); LV-proximal: AP: 1.0, L: 1.2, D: 2.3, n = 17; LV-intermediate: AP: 1.5, L: 1.3, D: 3, n = 7; and LV-distal: AP: 1.0, L: 2.1, D: 2.3, n = 17. Tumor growth was monitored weekly by bioluminescence following luciferin injection. For survival experiments, mice were maintained until reaching humane endpoint criteria following GBM xenograft. For histology analysis, mice were maintained for 4 weeks after tumor implantation (n=7). Mice were then anesthetized and perfused with 4% paraformaldehyde. Brains were extracted and cryoprotected in 30% sucrose. Brains were sectioned using an HM 430 Freezing Microtome at 30 μm thickness. Sections were stored in 30% ethylene glycol, 20% glycerol, 0.05M PBS, pH 7.4 at -20°C until immunohistochemical processing.

### Immunohistochemistry

Sections were permeabilized with 0.1% Triton in PBS (PBST) and blocked with 1% BSA and 10% normal horse serum. In the case of caspase-3 and Ki67 staining, antigen retrieval was performed using sodium citrate buffer (10 mM + 0.05% Tween) at 90°C for 25 minutes, followed by cooling in the sodium citrate buffer for 30 minutes before washing and blocking. Sections were then incubated overnight at 4°C in primary antibody at various concentrations (**Table 1**) diluted in 0.2% normal horse serum in PBST. Sections were washed and incubated in the dark for 1 hour at room temperature with secondary antibodies (**Table 2**) at a concentration of 1:500 in 2% normal horse serum in PBST. Sections were washed and counterstained with DAPI as a nuclear dye. At least three sections per animal were used per staining condition.

**Table 1:** Primary antibodies used.

Antibody	Species	Detection	Dilution Factor	Catalog
**GFP**	Mouse	GFP+ GBM cells	1:500	Abcam (ab1218)
**Human Nuclei (HuNu)**	Mouse	Human GBM cells	1:200	Millipore (MAB1281)
**Ki67**	Rabbit	Proliferating cells	1:200	Thermo (RM-9106-S0)
	Mouse			Novocastra (NCL-L-Ki67-MM1)
**phosphohistone H3 (pH3)**	Rabbit	Proliferating cells	1:200	Cell Signaling (9701S)
**GFAP**	Rabbit	Astrocytic cells	1:200	Dako (Z0334)
**SOX2**	Rat	Undifferentiated cells	1:500	Thermo (14-9811-82)
**OLIG2**	Rabbit	Oligodendrocyte precursors	1:500	Millipore (AB9610)
**Cleaved caspase-3 (Asp175)**	Rabbit	Apoptotic cells	1:200	Cell Signaling (9661)
**Doublecortin (DCX)**	Goat	Neuroblasts	1:200	Santa Cruz (SC-8066)

**Table 2:** Secondary antibodies used.

Secondary Antibody Wavelength	Species and Reactivity	Dilution Factor	Catalog
**Alexa Fluor 568**	Donkey anti-rabbit	1:500	Invitrogen (A10042)
**Alexa Fluor 647**	Chicken anti-rabbit	1:500	Invitrogen (A21443)
**Alexa Fluor 488**	Donkey anti-goat	1:500	Invitrogen (10246392)
**Alexa Fluor 555**	Donkey anti-mouse	1:500	Invitrogen (A31570)
**Alexa Fluor 594**	Donkey anti-rat	1:500	Invitrogen (A21209)

### Imaging

Immunohistochemical preparations were visualized using a confocal microscope (Zeiss LSM800). Tumors were visualized by GFP+ cells and imaged with 10X, 25X, 40X or 63X objectives. ZEN^®^ Blue Edition software (Zeiss) was then used to process the image. All sections for the same antibody combinations were imaged in the same way using the same exposure levels.

### Volumetric Analysis

Tumor area data was obtained using ZEN^®^ Blue Edition software. GFP+ tumors were traced using the “Draw Spline Contour” tool in ZEN software to obtain the area of each tumor section. Morphometric volume was then calculated using the Cavalieri principle, which allows an accurate estimation of the volume (V) of a structure independently of its shape and size (32). This is calculated by finding surface area (A) of a number (n) of parallel sections spaced at a constant distance (t) and inserting into the following equation: V = t * (A_1_ + A_2_ + A_3_… + A_n_).

### Cell Quantification

ZEN^®^ Blue software was used to estimate the number of cells expressing the human nucleus marker, HuNu, and the proliferation marker, Ki67 in the different groups, both in the tumor as in SVZ. In addition, cells positive for cleaved caspase-3, phosphohistone H3, SOX2, SOX2/GFAP, DCX and OLIG2 present in SVZ were quantified. For cell quantification of the SVZ, the ipsilateral and contralateral SVZ were imaged in 20X tiles using a confocal microscope. Using the ZEN^®^ Blue software, the SVZ region was manually traced using the “Draw Spline Contour” tool to specifically isolate the cells of the SVZ for the subsequent analysis. Signal background was removed by adjusting the channel histogram to the peak of the curve and remaining cells were considered positive and counted. Cell quantification was performed from planes with no tumor cell presence in order to avoid changes in cell proportion, The cell density (number of cells per square millimeter) was calculated for each image.

### Statistical Analysis

All data is represented as the mean ± the standard error (SEM) unless otherwise indicated. Statistical analysis and graphical representation were performed using GraphPad Prism^®^ 6 software. Normal distribution of data was assessed using the Shapiro-Wilk normality test. To compare among multiple groups, analysis of variance (ANOVA) with Tukey’s post-hoc correction was performed. For independent comparisons between two groups the student’s t-test was performed. The level of significance was determined as p < 0.05.

## Results

### GBM Proximity to the LV Contributes to Tumor Growth and Survival Outcome

**Figure 1:**
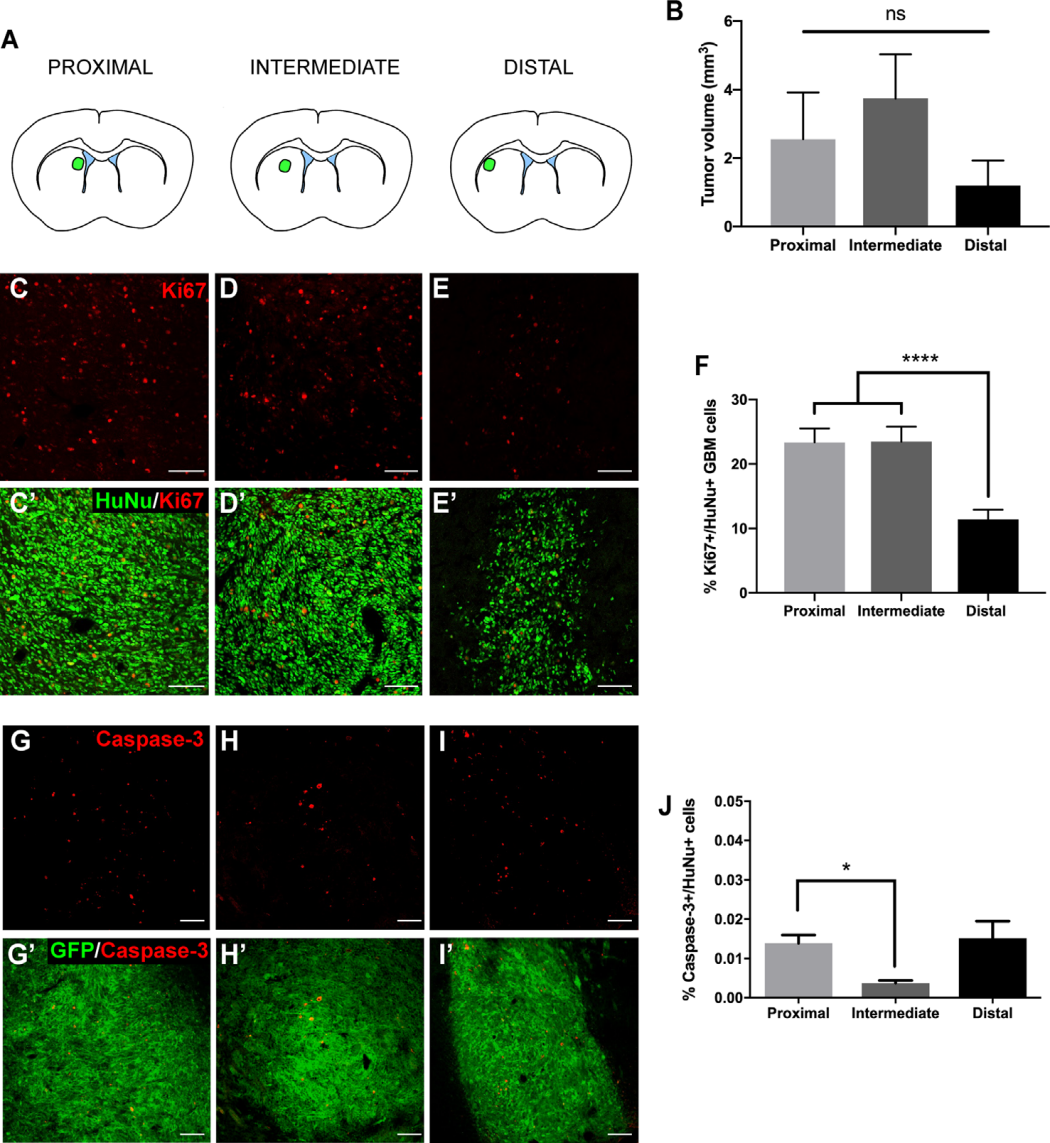
GBM proximity to the lateral ventricle induces increased tumor growth. **(A)** Schematic illustration of the LV-proximal, LV-intermediate, and LV-distal injection sites. **(B)** Quantification of GFP+ tumor volume in LV-proximal (n = 6), LV-intermediate (n = 5), and LV-distal (n = 5) groups. **(C–E)** Representative Ki67 immunohistochemical staining in **(C)** LV-proximal, **(D)** LV-intermediate, and **(E)** LV-distal GBM. Merged with HuNu staining (green, C’-E’). Scale bar = 100 μm. **(F)** Quantification of the percentage of Ki67+/HuNu+ cells within the GBM tumor between LV-proximal (n = 6), LV-intermediate (n = 5), and LV-distal (n = 5) groups. **(G–I)** Representative cleaved caspase-3 (cleaved C3) immunohistochemical staining in **(G)** LV-proximal, **(H)** LV-intermediate, and **(I)** LV-distal GBM. Merged with GFP staining (green, G’-I’). Scale bar = 100 μm. **(J)** Quantification of the percentage of cleaved caspase-3+/HuNu+ cells in the GBM tumor between LV-proximal (n = 7), LV-intermediate (n = 3), and LV-distal (n = 5) groups. The data are presented as mean ± SEM. *p < 0.05, ****p < 0.0001. NS, not significant.

We first evaluated the effect of the LV proximity on tumor growth in our animal model. Patient-derived GBM cells transduced to express GFP and luciferase were implanted at locations proximal, intermediate, and distal to the LV (**Figure 1A** and **Supplementary Figure 1**). Following a 4-week period, tumors were evaluated for volume, cellular density, proliferation, and apoptosis. When tumors were injected into locations proximal and intermediate to the LV, we observed a trend towards increased tumor volume compared to LV-distal tumors (LV-proximal: 2.55 mm^3^; LV-intermediate: 3.74 mm^3^; LV-Distal: 1.19 mm^3^; **Figure 1B**) with no difference in tumor cell density (**Supplemental Figure 2**). In order to determine whether LV proximity induced differences in proliferation or apoptosis, we performed immunofluorescence staining for Ki67, cleaved caspase-3, and human nuclei (HuNu)+ GBM cells. In tumors injected in LV-proximal and LV-intermediate locations we observed a significantly higher percentage of Ki67+/HuNu+ GBM cells than in LV-distal tumors (LV-proximal: 23.36%, LV-Intermediate: 23.48%, LV-Distal: 11.41%; **Figures 1C–F**), indicating an increase in GBM proliferative index dependent on proximity to the LV. Additionally, the percentage of cleaved caspase-3+/HuNu+ cells was significantly decreased in LV-intermediate tumors compared to LV-proximal tumors (LV-Proximal: 0.014%, LV-Intermediate: 0.0037%, LV-Distal: 0.015%; **Figures 1G–J**).

**Figure 2:**
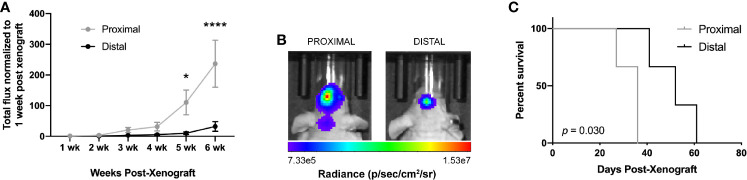
GBM proximity to the lateral ventricles impacts long-term outcome in rodents. **(A)** Quantification of BLI total flux fold change over time in the LV-proximal and LV-distal GBM tumor conditions (n = 7). **(B)** Representative BLI images in radiance (photons/second/centimeter/steradian) of immunosuppressed athymic nude mice bearing orthotopic patient-derived GBM at LV-proximal (left) and LV-distal (right) locations at five weeks post injection. **(C)** Kaplan-Meier survival curve of mice bearing tumors in LV-proximal and LV-distal sites (n = 3). The median survival for LV-proximal or LV-distal tumor bearing mice were 36 and 52 days, respectively. The data are presented as mean ± SEM. *p < 0.05, ****p < 0.0001.

We then evaluated the differences in tumor growth *via* bioluminescence imaging (BLI) and long-term survival outcome. Due to similar Ki67+ staining in LV-proximal and LV-intermediate tumor locations, we only used the LV-proximal tumor site for survival analysis. Tumor growth measured by increase in total flux (photons per second) was significantly higher in LV-proximal tumors than LV-distal tumors at 5 weeks post-xenograft (**Figures 2A, B**). Additionally, mice with LV-proximal tumors exhibited decreased median survival compared to their LV-distal tumor-bearing counterparts (LV-Proximal: 36 days, LV-Distal 52 days; **Figure 2C**). These findings show that we are able to effectively model several of the clinical differences of LV-proximal GBM compared to LV-distal GBM, such as increased tumor burden and decreased survival, in an immunocompromised rodent model.

### Proliferation Levels in GFP-/HuNu- Cells Within the SVZ Are Decreased as a Result of GBM Tumor Proximity

**Figure 3:**
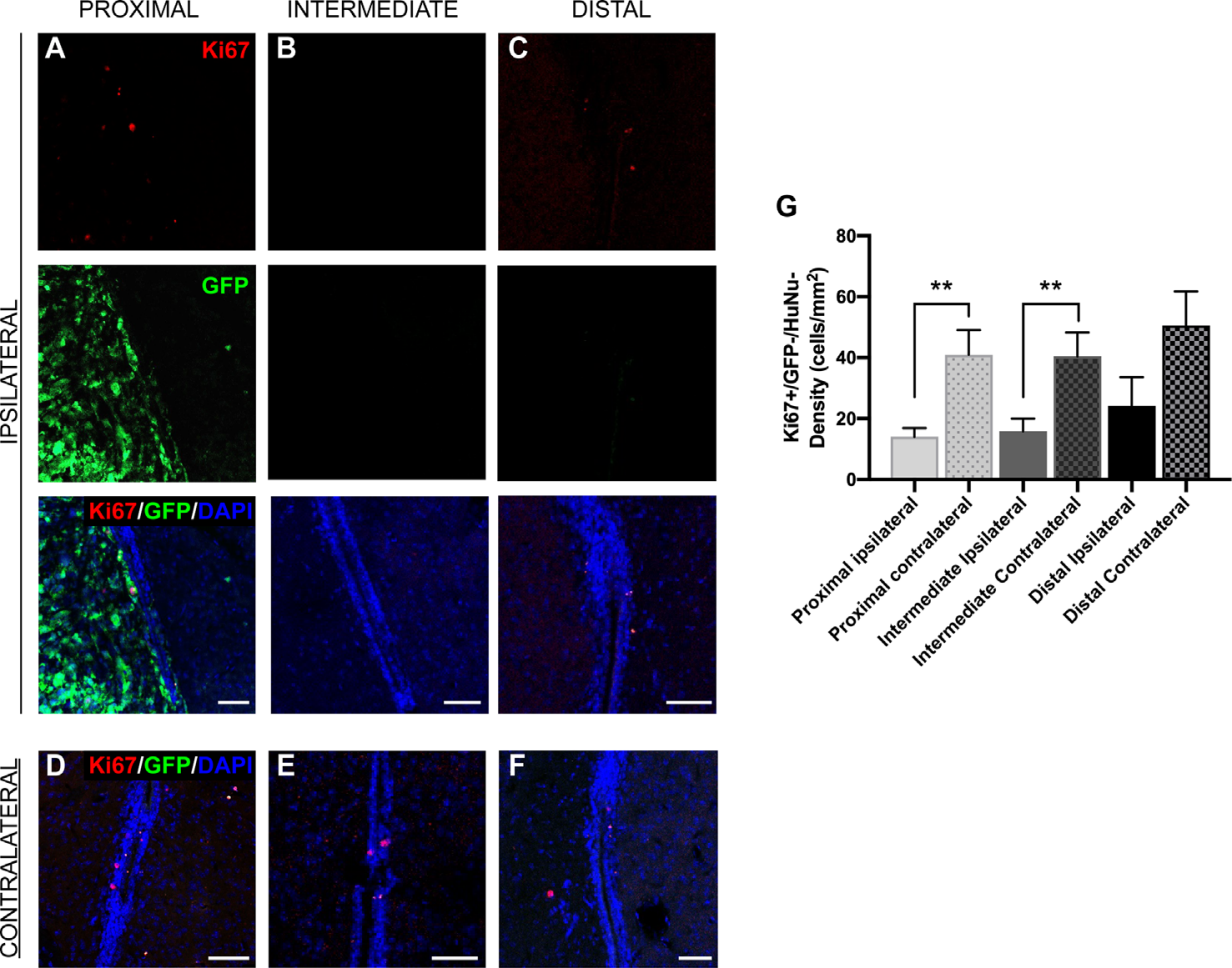
GBM proximity to the LV negatively influences Ki67 expression in the SVZ. **(A–F)** Representative images of immunohistochemical staining for Ki67 in GFP- cells of the SVZ **(A–C)** ipsilateral and **(D–F)** contralateral to the tumor. Red = Ki67, green = GFP, Blue = DAPI. This was compared between **(A, D)** LV-proximal, **(B, E)** LV-intermediate, and **(C, F)** LV-distal GBM groups. Scale bar = 50 μm. **(G)** Quantification of Ki67+ cell density in the SVZ comparing between the SVZ ipsilateral and contralateral to the GBM in LV-proximal (n = 6), LV-intermediate (n = 5), and LV-distal (n = 5) mice. Data are presented as mean ± SEM. **p < 0.01,

The LV contains the SVZ, the largest neurogenic niche in mammals (11–14). Previous studies have indicated that the cellular populations of the SVZ are altered due to the presence of GBM (26), but do not explore the effect of tumor proximity on different neurogenic cell populations. We observed that SVZ size was not altered by the presence of tumors when compared between groups and between sides ipsilateral and contralateral to the tumor (data not shown). To explore the effect of GBM proximity on mouse SVZ cell proliferation, we performed immunostaining for Ki67 and evaluated Ki67+/GFP-/HuNu- cells in the regions of the SVZ where the tumor growth was also present, in both ipsilateral and contralateral hemispheres. We determined that the proliferation rate of innate cells in the SVZ ipsilateral to the tumor site is significantly decreased compared to the contralateral SVZ in the presence of LV-proximal and LV-intermediate GBM, but not in LV-distal GBM (LV-proximal: 14.01 cells/mm^2^ ipsilateral *vs*. 40.97 cells/mm^2^ contralateral; LV-intermediate: 15.85 cells/mm^2^ ipsilateral *vs*. 40.41 cells/mm^2^ contralateral; LV-distal: 24.18 cells/mm^2^ ipsilateral *vs*. 50.60 cells/mm^2^ contralateral; **Figures 3A–G**), indicating that tumor proximity decreases SVZ cell proliferation.

To verify that SVZ cells have decreased levels of mitosis with increased tumor proximity, we also performed IHC for phosphohistone H3 (pH3), a marker of chromatin condensation with higher specificity for mitosis than Ki67. Again, we determined that the proliferation rate of HuNu- cells in the SVZ ipsilateral to the LV-proximal tumor is significantly decreased compared to the contralateral hemisphere (LV-proximal: 92.51 cells/mm^2^ ipsilateral *vs*. 182.93 cells/mm^2^ contralateral; **Supplemental Figures 3A–E**). In contrast, there was no decrease in the proliferation of the ipsilateral SVZ cells in LV-distal GBM when compared to the contralateral SVZ (LV-distal: 233.62 cells/mm^2^ ipsilateral *vs*. 214.67 cells/mm^2^ contralateral; **Supplemental Figures 3A–E**). Despite changes in proliferation, almost no cleaved caspase-3 labeling was seen in GFP- cells of the SVZ (data not shown). These findings further support a shift in the proportion of SVZ cell proliferation in response to tumor proximity.

### SOX2+/GFAP+/HuNu- Cells Within the SVZ Are Decreased as a Result of GBM Tumor Proximity

**Figure 4:**
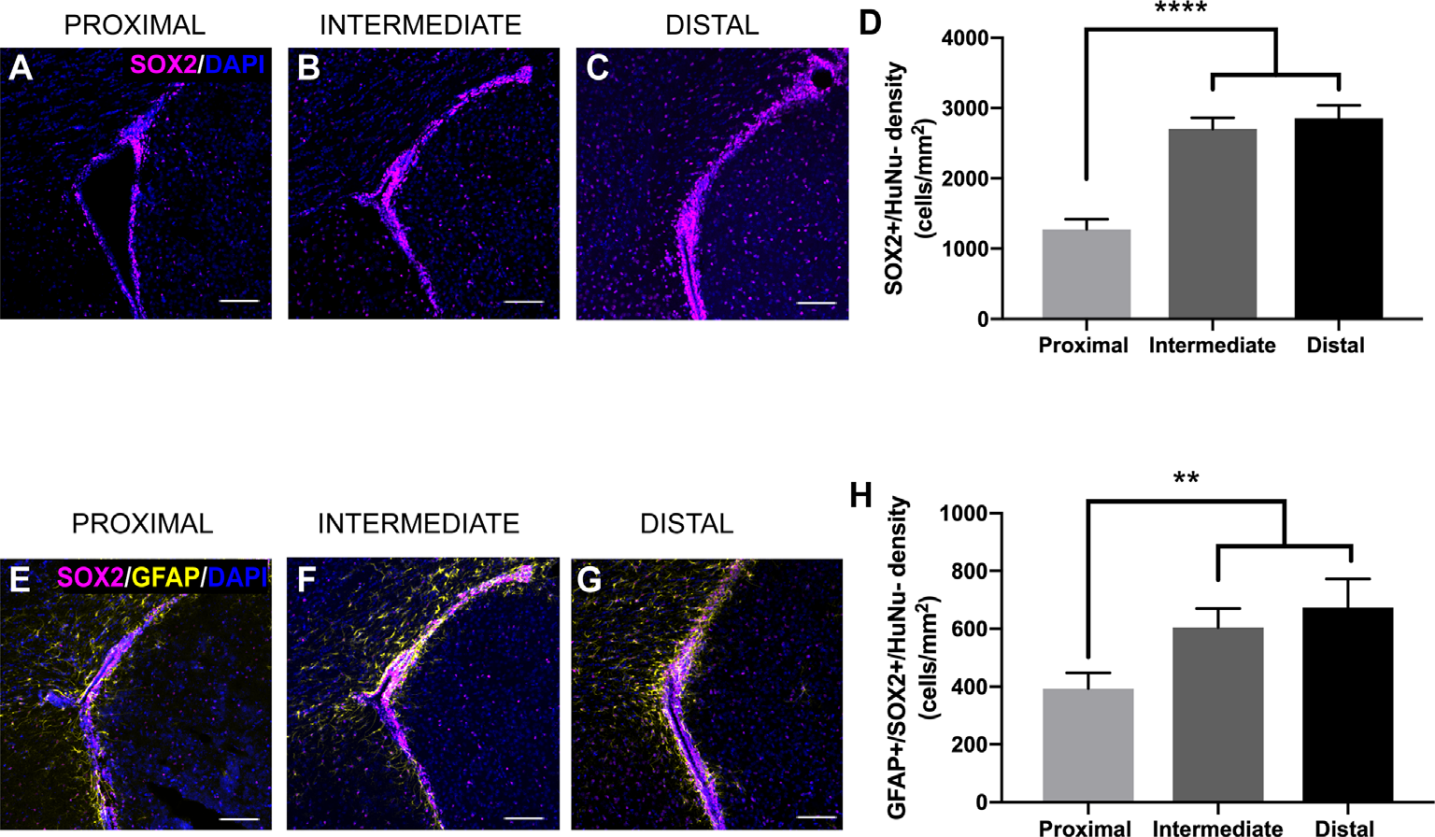
SVZ SOX2+ progenitor number is altered by LV-proximal GBM. **(A–C)** Representative images of SOX2+ cells in the GFP-/HuNu- cells of the SVZ in **(A)** LV-proximal, **(B)** LV-intermediate, and **(C)** LV-distal groups. Scale bar = 100 μm. **(D)** Quantification of SOX2+ cell density in the SVZ of LV-proximal, LV-intermediate, and LV-distal GBM mice. **(E–G)** Representative images of GFAP+/SOX2+ cells in the SVZ of **(E)** LV-proximal, **(F)** LV-intermediate, and **(G)** LV-distal groups. Scale bar = 100 μm. **(H)** Quantification of GFAP+/SOX2+ cell density in the SVZ of LV-proximal (n = 7), LV-intermediate (n = 5), and LV-distal (n = 5) GBM mice. Data are presented as mean ± SEM. **p < 0.01, ****p < 0.0001.

The SVZ contains NSCs that differentiate into progenitor cells, ultimately leading to the production of new neurons and glia throughout life (15–18). To examine how tumor proximity affects these populations of cells, we performed immunohistochemical staining for a variety of markers of different neurogenic cell types. We evaluated the staining for SOX2, a marker of NSCs and progenitors (33), in response to tumor proximity. SOX2+/HuNu- cell density is significantly decreased in the ipsilateral SVZ of LV-proximal tumors compared to LV-intermediate and LV-distal tumors (LV-proximal: 1273.83 cells/mm^2^; LV-intermediate: 2706.12 cells/mm^2^; LV-distal: 2853.37 cells/mm^2^; **Figures 4A–D**). Cells that are positive for both GFAP and SOX2 and negative for HuNu, that represent astrocytic NSCs of the SVZ (34), were also decreased in response to LV-proximal tumors compared to LV-intermediate and LV-distal tumors (LV-proximal: 393.20 cells/mm^2^; LV-intermediate: 604.48 cells/mm^2^; LV-distal: 673.43 cells/mm^2^; **Figures 4E–H**), showing that there is a decrease in NSCs and progenitors in the SVZ in response to LV-proximal tumors.

### GBM Proximity to the Lateral Ventricle Decreases Oligodendrocyte Precursor and Neuroblast Density in the SVZ

**Figure 5:**
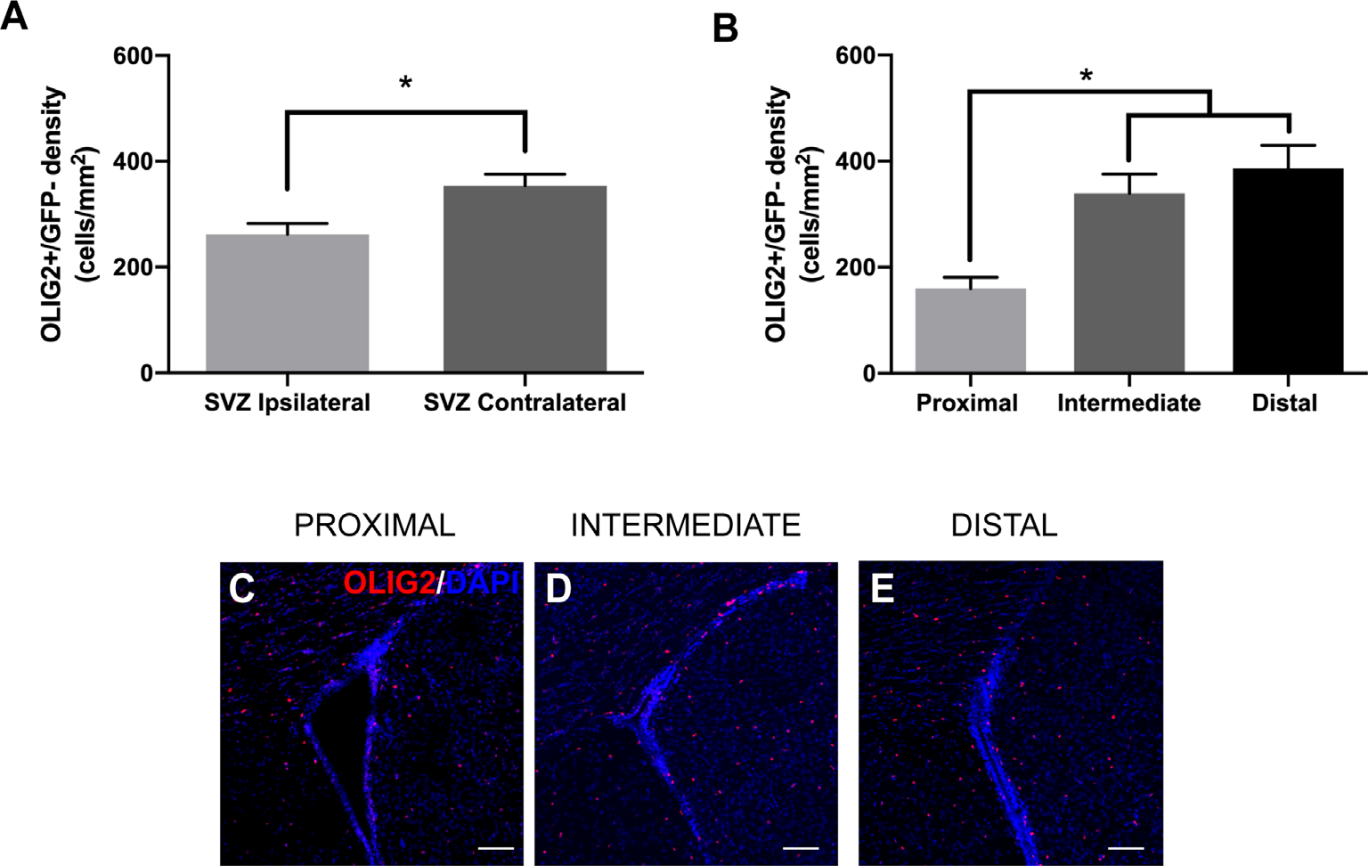
LV-proximal GBM reduces the number of OLIG2+ progeny in the ipsilateral SVZ. **(A)** Quantification of OLIG2+ cell density in the ipsilateral and contralateral SVZ of all groups. **(B)** Quantification of OLIG2+ cell density in the SVZ ipsilateral to the tumor in LV-proximal, LV-intermediate, and LV-distal GBM mice. **(C-E)** Representative images of the ipsilateral SVZ in **(C)** LV-proximal (n = 7), **(D)** LV-intermediate (n = 5), and **(E)** LV-distal (n = 5) groups. Scale bar = 100 μm. Data are represented as mean ± SEM. *p < 0.05.

To examine changes in neurogenic progeny in the SVZ, we also analyzed the number of GFP-/oligodendrocyte precursor cells (OPCs) and neuroblasts in relation to GBM tumor proximity. The differentiation of NSCs to OPCs is accompanied by the expression of the transcription factor OLIG2. We found that the presence of GBM significantly decreases the number of GFP-/OLIG2+ cells in the ipsilateral SVZ compared to the contralateral SVZ among all groups (ipsilateral 261.95 cells/mm^2^ *vs*. contralateral 353.96 cells/mm^2^; **Figure 5A**). Additionally, there are significantly fewer GFP-/OLIG2+ cells in the ipsilateral SVZ of LV-proximal group than in the LV-intermediate or LV-distal groups (LV-proximal: 159.88 cells/mm^2^; LV-intermediate: 339.27 cells/mm^2^; LV-distal: 386.19 cells/mm^2^; **Figures 5B–E**), indicating that GBM proximity to the SVZ significantly decreases OPC generation in the SVZ.

**Figure 6:**
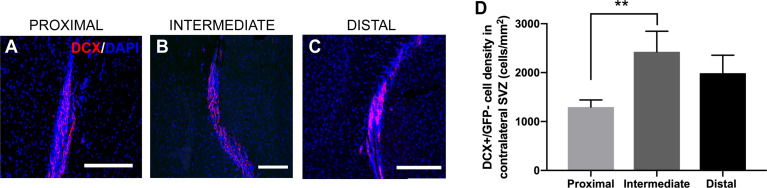
LV-proximal GBM decreases the number of neuroblasts in the contralateral SVZ. **(A–C)** Representative images of DCX+ cells in the contralateral SVZ to the GBM in **(A)** LV-proximal (n = 7), **(B)** LV-intermediate (n = 3), and **(C)** LV-distal (n = 5) groups. Scale bar = 100 μm. **(D)** Quantification of DCX+ in the contralateral SVZ to the tumor between groups. Data is presented as mean ± SEM. **p < 0.01.

The differentiation of NSCs into neuroblasts, as well as their migration through the brain and incorporation into the olfactory bulb, is well-studied in rodents (16, 35). Previous studies have shown an increase in the levels of SVZ neuroblasts in the presence of GBM, as well as neuroblast migration to the tumor site (26). In order to examine how the neuroblast population was changed in response to tumor proximity to the SVZ, we performed immunohistochemical staining for doublecortin (DCX+), a widely used marker for migratory neuroblasts. Interestingly, there was no significant change in GFP-/DCX+ cells among groups when measuring in the SVZ ipsilateral to the injection site (LV-proximal: 1608.86 cells/mm^2^; LV-intermediate: 1936.69 cells/mm^2^; LV-distal: 2484.84 cells/mm^2^; **Supplemental Figure 4**), although there was a trend towards decreased GFP-/DCX+ cells with increased GBM proximity to the LV. These findings suggest that the proximity of GBM to the LV does not affect NSC differentiation down the neuroblast lineage, despite changes in the number and proliferation of NSCs. We did not observe any GFP-/DCX+ cells migrating to the tumor site (data not shown). While there were no significant changes in the ipsilateral hemisphere to the tumor, mice with LV-proximal tumors had significantly decreased DCX+ cells in the contralateral hemisphere than LV-intermediate conditions (LV-proximal: 1295.65 cells/mm^2^; LV-intermediate: 2427.31 cells/mm^2^; LV-distal: 1988.12 cells/mm^2^, **Figures 6A–D**).

## Discussion

In this study, we highlight a two-way relationship between GBM tumors and SVZ biology dependent on tumor proximity to the LV in rodents. Our results indicate that human GBM cells respond to the LVs in a proximity-dependent manner by increasing their proliferation, ultimately resulting in decreased survival. Furthermore, we observed that tumor proximity to the LV decreases some aspects of neurogenesis in the SVZ, including proliferation as well as the density of NSCs and progenitors.

GBM tumors are more malignant in patients when located proximal to the LV than in LV-distal counterparts. The increased malignancy is evidenced by increased tumor size, increased distal recurrence, and decreased survival independent of extent of resection (5, 6, 8, 9, 36). Our work is the first to study the proximity-dependent interaction between GBM and the SVZ in a rodent model. This model recapitulates several of the features of LV-proximal GBM in patients, including increased tumor growth, increased proliferation, and decreased survival. It remains unclear the reason for increased malignancy in these tumors. Despite previous studies describing worse prognosis in patients with GBM close to the LVs, there is no substantial evidence tying these clinical findings to a molecular signature of GBM. Although some studies have linked LV-proximal GBM to characteristics such as molecular subtype and the expression of stem cell markers, others have found no association of LV-proximal GBM with a molecular signature (37–39). This may indicate that the increased malignancy of LV-proximal GBM may not be a cell-intrinsic factor, but a product of the SVZ microenvironment. This is supported by our previous studies as well as this work, where tumors derived from the same cell line become more malignant in response to the LV microenvironment.

Though this work does not identify the components responsible for increased malignancy in these tumors, there are several potential sources of neurogenesis-supporting factors that may contribute to GBM growth. The SVZ contains many NSC and progenitor cells that may interact directly with GBM cells, thereby increasing proliferation. Additionally, the SVZ contains many soluble factors which contribute to neurogenesis of SVZ NSCs that GBM cells may take advantage of. These factors may be released from the NSCs themselves or be contained within the nearby cerebrospinal fluid (CSF), ultimately contributing to GBM malignancy (40–43). Our previous work has revealed several CSF-induced transcriptomic changes in primary GBM cells, including upregulation of SERPINA3, MYC, and SPP1 (40, 41). Gene ontology analysis has indicated an upregulation in cell viability, movement, and migration pathways induced by CSF (40), all of which may contribute to the malignancy-promoting pathways in LV-proximal tumors. These *in vitro* findings warrant the study of transcriptomic changes in SVZ and GBM cells *in vivo* using a model similar to the one presented here. The elucidation of the bidirectional mechanisms supporting GBM tumor growth requires further unbiased transcriptomic studies in both animal models and navigation-guided tumor biopsy samples.

We observed decreased proliferation of SVZ cells in the presence of LV-proximal and LV-intermediate GBM tumors compared to LV-distal tumors. These findings agree with previous work, which found decreased proliferation of SVZ cells in the presence of GBM in an syngeneic intracranial C6 rat glioma model (26). However, other studies have found that signaling from the tumor increases SVZ proliferation, resulting in hypertrophic, hypercellular areas and increased levels of stem cell markers such as Nestin (44). One potential reason for these contradictive findings is a different mechanism of interaction by GBM cells and resident SVZ cells dependent on tumor proximity to the LV. Soluble factors that are expressed by GBM, such as PDGF-A, have the ability to increase the levels of proliferation in NSCs, resulting in hypertrophic areas of the SVZ that share some features of gliomas (45, 46). However, a different effect is seen when wild-type NSCs are directly placed in co-culture with *Ink4a/Arf-/-*, EGFRvIII mutated NSCs that generate tumors *in vivo* which recapitulate many features of human GBM (47, 48). Here, direct contact with glioma-like cells results in decreased levels of proliferation and increased levels of quiescence in NSCs primarily mediated by increased Notch signaling activation (48). This suggests that GBM proximity to the LV may differentially affect resident NSCs, where LV-proximal tumors induce decreased proliferation and increased quiescence through cell-cell contact *via* Notch signaling, while LV-distal tumors may signal to NSCs primarily through secreted components.

The SVZ of mice with LV-proximal tumors have decreased neurogenic capability compared to those bearing LV-intermediate or LV-distal tumors, measured through decreased SOX2+ stem cells and progenitors, decreased SOX2+/GFAP+ NSCs, and decreased numbers of OLIG2+ cells in the ipsilateral SVZ. Though there is no significant difference in the numbers of DCX+ neuroblasts in the ipsilateral SVZ among groups, there is also a decrease in DCX+ neuroblasts in the contralateral SVZ of LV-proximal tumor mice compared to LV-intermediate tumor mice, suggesting decreased neurogenesis in the contralateral hemisphere of LV-proximal mice. Interestingly, previous data shows that GBM tumors increase neuroblast density in the SVZ (26), which differs from our present findings. SOX2+ NSCs are able to give rise to new neural cells through their multipotent potential (49). Therefore, by decreasing the number of stem cells or the rate and number of cell divisions, it is expected that we will find a lower rate of neuronal renewal in SVZ, which is a direct alteration in neurogenesis (50). Decreased neurogenesis in the ipsilateral SVZ may be due to the previously mentioned increase in NSC quiescence *via* Notch signaling through cell-cell contact. Increased quiescence of NSCs results in both decreased proliferation and decreased differentiation into progenitors (51). The decrease of neuroblasts in the contralateral hemisphere, however, may suggest the secretion of a circulating factor that is able to affect SVZ neurogenesis in the hemisphere contralateral to the tumor. The identification of factors that decrease SVZ neurogenesis secreted by GBM cells or other cells in response to the presence of GBM, such as ependymal cells or cells of the choroid plexus, need to be further explored.

Alternatively, the decrease of neuroblasts in the contralateral hemisphere may be related to decreased CSF volume or flow throughout the ventricular system without directly altering secreted factors. Neurogenesis is regulated in part by both the flow and the contained chemokines within the CSF. Both the proliferation of NSCs and the migration of newly differentiated neuroblasts down the rostral migratory stream are regulated in a flow-dependent manner (43, 52). The decrease of CSF flow and the loss of chemokine gradient may affect neurogenesis, particularly stem cell proliferation and neuroblast migration. In support of this scenario, glioma-bearing mice have reduced CSF circulation and output compared to non-tumor controls (53), which could implicate a loss of flow-dependent regulation in GBM. The contribution of CSF flow to neurogenesis and GBM malignancy in this animal model require further studies to fully understand.

Interestingly, there are significant decreases in the level of cleaved caspase-3 labeling in the tumors LV-intermediate tumor group. GBM tumors have quite low levels of caspase-3 labeling in humans (54), so further decrease in apoptosis of the tumor may be related to increased growth. This, accompanied by increased Ki67+ GBM cells, may indicate that LV-intermediate tumors were located in a “sweet spot” where the tumors are able to take advantage of neurogenic factors contained within the SVZ niche without leading to significant neurogenic disruption. The signaling pathways between the SVZ and GBM that regulate cell proliferation and apoptosis need to be further studied in order to determine the molecular contributors to this phenomenon.

In summary, this study provides the development of a novel rodent model of LV-proximal GBM. Due to the limitations of using human cells in an immunocompromised rodent model, it will be necessary to further evaluate and validate these observations in immunocompetent murine models. The proximity of the tumor to the LV results in increased tumor proliferation, increased tumor growth, and decreased survival. Additionally, we have determined that GBM proximity to the LV also negatively impacts the number of NSCs and downstream progenitors in the SVZ. This model will be invaluable for future studies to describe the interactions between the SVZ and GBM tumors, as well as for the investigation of novel therapeutics to target signaling between these two sites. Ultimately, these findings encourage future studies to elucidate the bidirectional molecular signaling between GBM and the SVZ, particularly the identification of pathways contributing to tumor progression in LV-proximal GBM patients.

## Data Availability Statement

The raw data supporting the conclusions of this article will be made available by the authors, without undue reservation.

## Ethics Statement

The animal study was reviewed and approved by Institutional Animal Care and Use Committee of Mayo Clinic.

## Author Contributions

EN, CR-P, and HG-C, conceptualized, lead the project, and analyzed the data. LR, EN, RB-V, SJ, ML-V, AC, NZ, CV-R, CR-P, and HG-C performed the experiments and analyzed the data. AQ-H performed tissue collection. HG-C provided funding. All authors contributed to the article and approved the submitted version.

## Funding

EN was supported by the Mayo Clinic Graduate School of Biomedical Sciences, the Mayo Clinic Center for Regenerative Medicine, and the Uihlein Professorship Research Grant. ML-V was supported by CONACYT, UNAM and the Uihlein Professorship Research Grant. AQ-H was supported by the Mayo Clinic Professorship and a Clinician Investigator grant and the National Institutes of Health (NIH) (R43CA221490, R01CA200399, R01CA183827, R01CA195503, and R01CA216855). HG-C was supported by NIH grants (R03NS109444, R21CA221490, and K01NS11093001).

## Conflict of Interest

The authors declare that the research was conducted in the absence of any commercial or financial relationships that could be construed as a potential conflict of interest.
