# Supplementary material for: Glioblastoma Proximity to the Lateral Ventricle Alters Neurogenic Cell Populations of the Subventricular Zone
Source: Front Oncol. 2021 Jun 29;11:650316. doi: 10.3389/fonc.2021.650316 (PMC8277421; doi:10.3389/fonc.2021.650316)
Supplement: Supplementary file 1 [file Image_1.pdf]

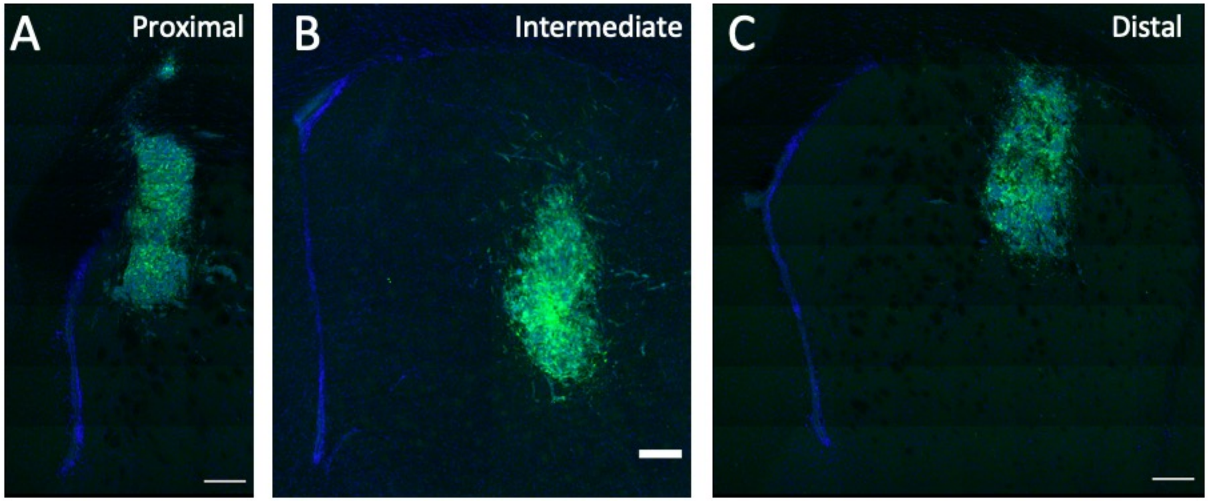

Supplemental Figure 1: Panoramic examples of tumor injection sites. (A): Proximal, (B): Intermediate, and (C): Distal. Scale bar = 200 μm.

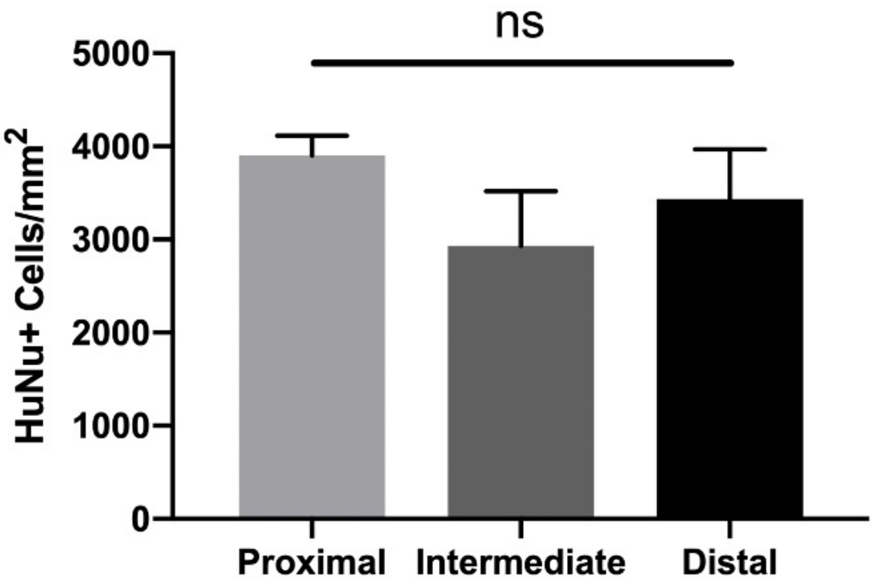

Supplemental Figure 2: Tumor cell density is not affected by GBM proximity to the LV in rodents. Quantification of HuNu+ cells per square mm within the GFP+ tumor bulk compared between LV-proximal, LV-intermediate, and LV-distal GBM. Data is presented as mean ± SEM.

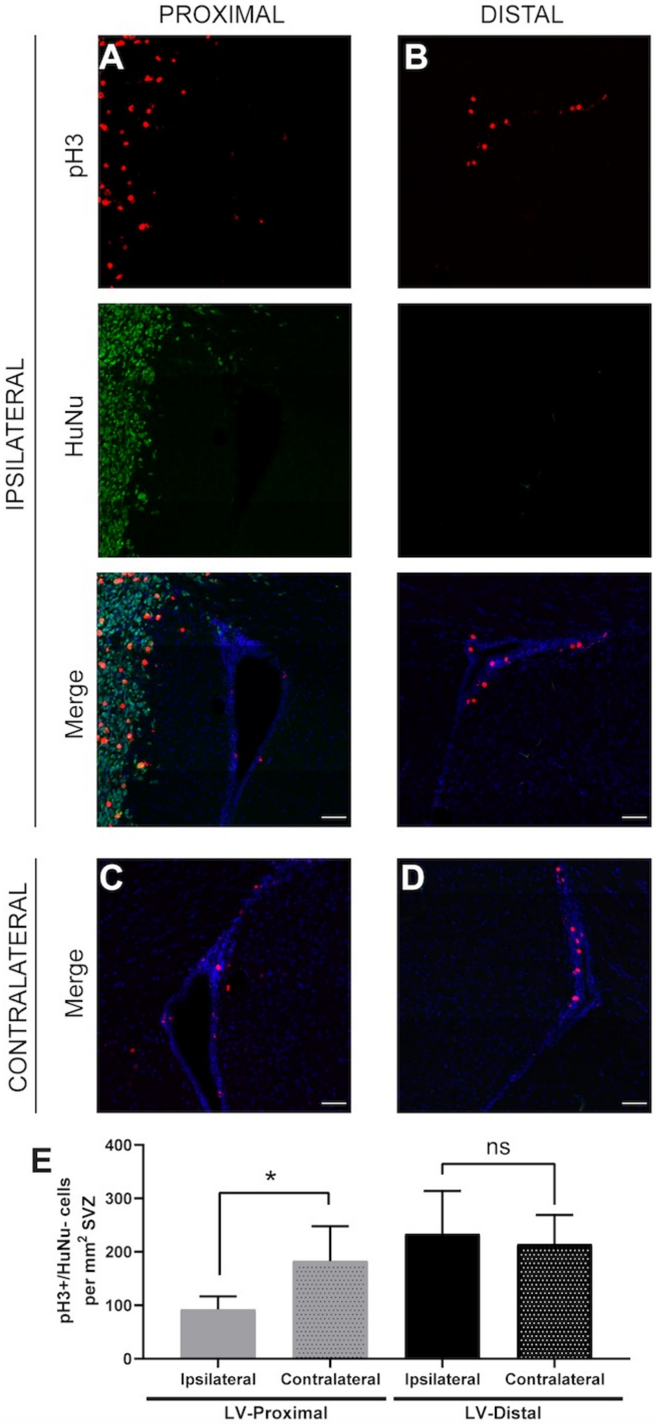

Supplemental Figure 3: GBM proximity to the LV negatively influences the number of pH3+/HuNu- proliferating cells in the SVZ. (A-D) Immunohistochemical staining for pH3 in HuNu- cells of the SVZ (A, B) ipsilateral and (C, D) contralateral to the tumor. Red = pH3, green = HuNu, Blue = DAPI. This is compared between (A, C) LV-proximal and (B, D) LV-distal GBM groups. Scale bar = 50 um. (E) Quantification of pH3+ cell density in SVZ comparing SVZ ipsilateral and contralateral to the GBM in LV-proximal and LV-distal mice. Data are presented as mean ± SEM. * $p < 0.05$

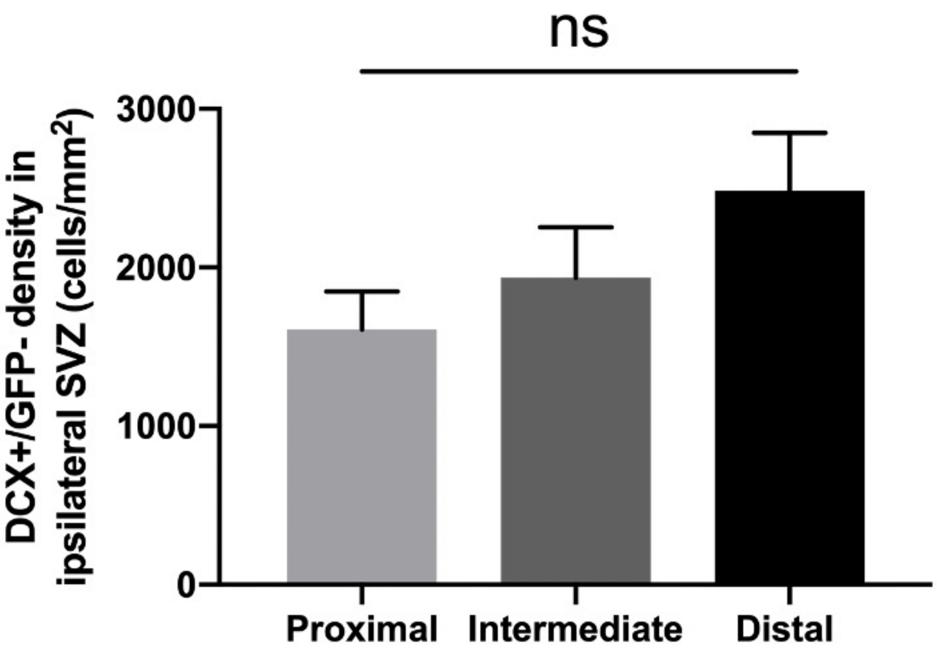

Supplemental Figure 4: Ipsilateral SVZ neuroblast density is not affected by GBM proximity to the LV in rodents. Quantification of DCX+ cells per square mm within the ipsilateral SVZ to the tumor compared between LV-proximal, LVintermediate, and LV-distal GBM. Data is presented as mean ± SEM.
